# Parents’ Inadequate Estimate of Their Children’s Objectively Physical Activity Level

**DOI:** 10.3390/children9030392

**Published:** 2022-03-10

**Authors:** Karin Kippe, Adilson Marques, João Martins, Pål Arild Lagestad

**Affiliations:** 1 Department of Teacher Education and Art, Nord University, 7600 Levanger, Norway; karin.o.kippe@nord.no; 2 CIPER, Faculdade de Motricidade Humana, Universidade de Lisboa, 1649-004 Lisboa, Portugal; amarques@fmh.ulisboa.pt (A.M.); jmartins@fmh.ulisboa.pt (J.M.); 3 Centro de Estudos em Educação, Faculdade de Motricidade Humana e UIDEF, Instituto de Educação, Universidade de Lisboa, 1649-004 Lisboa, Portugal

**Keywords:** parents’ estimation, physical activity, children, accelerometer, leisure

## Abstract

This study aimed to investigate parents’ estimation of their preschool children’s leisure-time physical activity (PA) and the correlation between parents’ reported participation in PA with their children in leisure time and their children’s PA levels. A total of 244 Norwegian preschool children aged 4–6 and their parents were enrolled in the study. According to standard protocols, the children’s PA level was measured with Actigraph GT1M accelerometers. The parents completed a questionnaire that provided information about their estimation of their children’s PA and their reported participation in their children’s PA. Correlation analyses and scatter plots showed no significant association between parents’ estimation of their children’s PA level at leisure time and the children’s objectively measured PA level. Only 5% of the parents estimated their children’s PA level correctly. In general, the parents overestimated their children’s PA levels by three times. Furthermore, the results found no significant correlation between children’s PA levels at leisure time and parents’ reported participation in PA with their children. Our findings indicate that parents’ self-estimation of their children’s PA is inaccurate, which is problematic. Considering that the PA levels of many children are too low to fulfill internationally established health recommendations, parents’ ‘wrong’ perception about their children’s PA urgently needs to be addressed and rectified.

## 1. Introduction

Physical activity (PA) substantially reduces the risk of illness, improves the quality of life, and increases functional ability [1]. Moreover, PA strengthens muscles and the skeleton, develops motor skills, reduces anxiety and depression, bolsters self-confidence, and contributes to social interaction [2]. PA also reduces the risk of cardiovascular disease in children significantly [3,4,5]. Therefore, it is recommended that children engage in moderate to vigorous PA (MVPA) for, on average, a minimum of 60 min per day [6]. However, research has shown that not all children satisfy these health recommendations for PA [7,8,9,10]. In addition, a cross-sectional study reports that the total amount of PA decreased by an average of 4.2% each year from the age of 5 to 18 [11]. Such findings have become a legitimate cause of great concern, taking into account that physical inactivity is a leading risk factor for global mortality [12], and inactivity among children and youth has been linked with lower PA levels in later life [13].

Almost all Norwegian children from three- to six years old are in preschool most of their waking hours [14]. Thus, preschool constitutes the main arena for children’s PA. However, extant literature provides evidence that children do not meet the health recommendations for PA during preschool time [15,16,17,18].

Previous longitudinal studies and other researchers point out that lifestyle behavior will follow the same trend from preschool to adulthood [13,19,20]. Furthermore, children are mostly in relationships with significant others, such as family members. Children internalize and take possession of their families’ attitudes, values, and actions through interaction, closeness, and identification [21]. Therefore, parents influence children’s physical capital. In leisure time, parents are responsible for reducing their children’s sedentary time and increasing PA. Parents’ assessment of their preschool children’s PA is thus of crucial importance. Several investigations report that adults overestimate their PA level and that of their children [22,23,24,25]. Moreover, in a study, parents with girls were more likely to overestimate their daughters’ activity level [26]. The same study showed that parental support for their children’s PA (transport, encouragement, and participation with the child) was positively associated with higher overestimation [26]. The authors highlight that parental PA support was positively correlated with children’s PA. Indeed, providing more support appears to be associated with higher overestimation.

It is suggested that PA should be prompted within the first five years of life because children’s activity patterns are relatively more easily influenced and open to changes and adoptions during this developmental period [27,28]. Dowda et al. [29] identified a significant direct relationship between a child’s MVPA and family support, including the variable of PA participation with the child. In a longitudinal study, Cleland et al. [30] found that mothers were important role models for PA and that fathers’ reward (praise for participation in PA) and direct support (bringing to and from activities, payment for participation and equipment) were the factors that affected PA most positively. For girls, active participation of the mother and sibling(s) was most important for PA in MVPA [30]. Of the family environment features assessed in the study of Tandon, Saelens, and Copeland [31], maternal and sibling co-participation in PA was directly associated with the average change in MVPA among girls. Tandon et al. [31] reported that having a family member that participates in PA with girls, rather than observing or receiving support or praise from other family members, might be an important factor for promoting PA. Several investigations supported the assertion that the children’s mothers’ PA level is a direct link to their children’s PA [32,33,34,35].

In free-living, where children are in free natural play and activity situations, the accuracy of the parent- or self-reported measurements of PA is often regarded as unreliable as a result of either overreporting or underreporting [36]. We argue that it is difficult to estimate children’s PA intensity levels without using an objective instrument, such as an accelerometer. Previous literature has not used objective measurements of preschool children’s PA. Addy et al. [37] also recommended using objective measurement instruments to capture children’s intermittent movement patterns better. Children tend to have relatively limited abilities to remember activities that they have performed.

The two main aims of this study are: (1) to elucidate the association between parents’ self-report of their children’s PA and their children’s objectively-measured PA level; and (2) to examine the relationship between parents’ self-reported participation in their children’s PA and the children’s objectively-measured PA.

## 2. Material and Methods

### 2.1. Subjects and Procedures

A total of 13 preschools in the northern part of Troendelag, a county located in central Norway, were randomly selected to participate in the study. The county consists of both cities and more rural areas. All of the 13 preschools in the study were located on the city’s outskirts. The eligibility criteria of this study were that all children had to be full-time preschoolers and 4–6 years of age. Out of the 13 preschools, 364 children met both criteria, of which 289 children volunteered to participate in the study with the approval of their primary guardians. Out of these, 244 children (125 boys and 119 girls) had valid accelerometer data, 213 mothers and 167 fathers completed the parental questionnaire, and 122 children had valid MVPA data and both of the parents’ estimation data. Because of the randomly selected participants, the children represented families with varied socioeconomic status according to income and education levels. Because of this, most of the parents were ethnical Norwegians, while approximately 18% of the children had parents born outside Norway. The families mostly lived in rural areas, with some families living in small Norwegian Towns (approximately 10,000 residents). All families had available areas for play and PA close to the house. Before the data collection and providing written informed consent, parents were given written and oral information about the procedures and ethical standards for testing related to sports science. This included information about the participants’ right to decline to participate and their right to withdraw from the research at any time once it has started. The study was approved by NSD (Norwegian Centre for Research Data, ref. 52221), Norway’s main research ethics committee.

### 2.2. Accelerometers

PA was measured with standardized procedures used in a large population study among children in Norway [18]. PA data were objectively collected using Actigraph GT1M accelerometers (ActiGraph, Fort Walton Beach, FL, USA). Accelerometers have emerged to be a valid and reliable tool for measuring daily PA and intensity among children [38,39,40,41,42] and the extent of fulfillment of international health recommendations [43]. Activity that produced 100–1999 counts per minute (CPM) was interpreted as light PA, whereas activity with less than 100 CPM was defined as sedentary. The moderate activity required 2000–5998 CPM, whereas activities that produced more than 5998 CPM were interpreted as vigorous PA [18].

Subjects were required to wear the accelerometers for seven consecutive days as recommended [37,44,45]. The data collection was carried out in May 2017, under normal weather conditions (temperature 10–20 °C, without snow, with the normal wind (0–5 m/s) and not much rain (0–4 mm of precipitation daily)), according to weather data. In order to ensure that all subjects wore the device throughout the period, a text message was sent to the parents each morning, reminding them to have their child wear the accelerometer. The parents were also informed about the accelerometer’s use and that it had to be placed on the right hip. The subjects had to wear the accelerometer during all waking hours, except during showering or other water activities. The accelerometers were set to start recording at 06:00 a.m., the day after they were distributed and put on, as an attempt to counteract the Hawthorne effect [46].

At the end of the data collection, the accelerometers were retrieved from the subjects. The data were downloaded to the ActiLife v6 13.3 software program (ActiGraph LLC, Fort Walton Beach, FL, USA). According to the protocols of Kolle et al. [18], the accelerometers recorded data in 10 s epochs. They provided the number of counts every 10 s for the 7-day period. Subjects were required to have at least two valid days to be included in the analyses. For data to be considered valid, at least 480 min of daily recorded activity was necessary [18]. Additionally, sequences of 20 min or more with zero counts were interpreted as non-wearing time and therefore omitted. Due to instructions stating that subjects were not to wear accelerometers during sleep at night, data between 00:00 a.m., and 05:59 a.m. were also excluded. Wear-time was categorized into the following variables: preschool hours (08:00 a.m.–03:29 p.m.); leisure time on weekdays (06:00 a.m.–07:59 a.m. and 03.30 p.m.–11:59 p.m.); and leisure time on weekends (06:00 a.m.–11:59 p.m.). These operationalizations were made based on feedback from parents and preschool staff, who identified these times as time spent in preschool and leisure, respectively.

### 2.3. Questionnaires

The parents with the responsibility for the daily care of the children (no data were collected on the biological relationship between the parent and child) were given a questionnaire that they were asked to complete to provide relevant information, including their estimation of their children’s minutes of PA in MVPA at leisure time, and their participation in percentage in their children’s PA at leisure time. The response format to the survey questions was open ended and they were asked to write a number from 0 to 100. These questions were: “When your 4–6-year-old children is in PA, how many percent of this time will you estimate you in average participate with your child?”, and “the health recommendations highlight that children should participate in PA where they are being warm and sweaty 60 min a day. How many minutes do you estimate your 4–6-year-old child is in such activity per on an average day at leisure, outside preschool?”

### 2.4. Data Analysis

The accelerometer and questionnaire data were analyzed in SPSS Statistics version 26 (IBM SPSS, Chicago, IL, USA). Descriptive characteristics were presented with mean and standard deviation. Bivariate Pearson correlation analysis was used to identify the association between children’s MVPA and parents’ estimates of their children’s PA level and participation in their children’s PA, respectively. Independent t-tests were employed to examine differences of the children where both parents had reported their estimation level (122 children) and the group of children with missing data from the mother, the father, or both (122 children). This is to examine whether the dropout was randomly and if it affected the PA level of the children. To visualize the association between parents’ estimations and children’s objectively-measured PA, scatter plots were used. Statistical significance was set at *p* ≤ 0.05.

## 3. Results

Table 1 shows that in one-half of the total parent participants, both parents estimated their children’s PA level in leisure. In addition, more mothers than fathers estimated their children’s activity level. Finally, the gender of the children did not affect the parents’ response rate.

The descriptive characteristics of the variables included in the study are presented in Table 2. Statistical analyses show no significant differences between the results of children with both parents’ estimation level (122 with all data), and the results of the group without data from both the children, the mother, and the father (*p* > 0.05).

The analyses showed no significant association (*p* > 0.05) between mothers’ and fathers’ estimation of their children’s PA and their children’s objectively-measured PA (Table 3). Furthermore, the analyses showed no significant association (*p* > 0.05) between parents’ participation and estimation and the PA level of children between the 122 participants with all data and the 122 participants with missing data.

**Figure 1:**
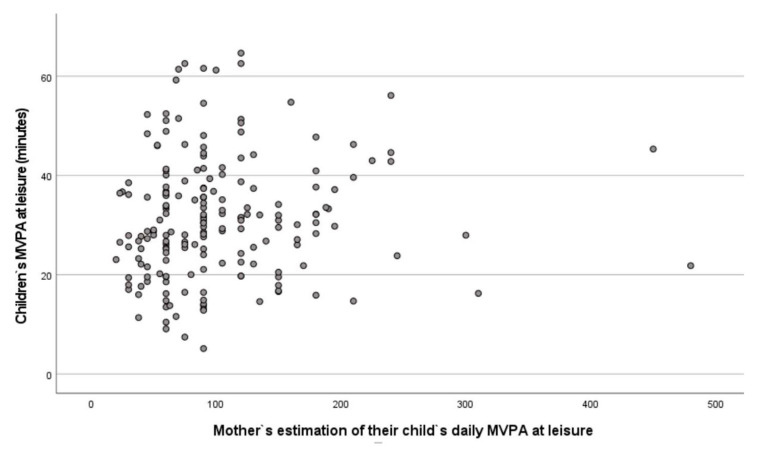
Scatter plot of preschool children’s MVPA at leisure time and mothers’ estimation of their children’s PA level.

**Figure 2:**
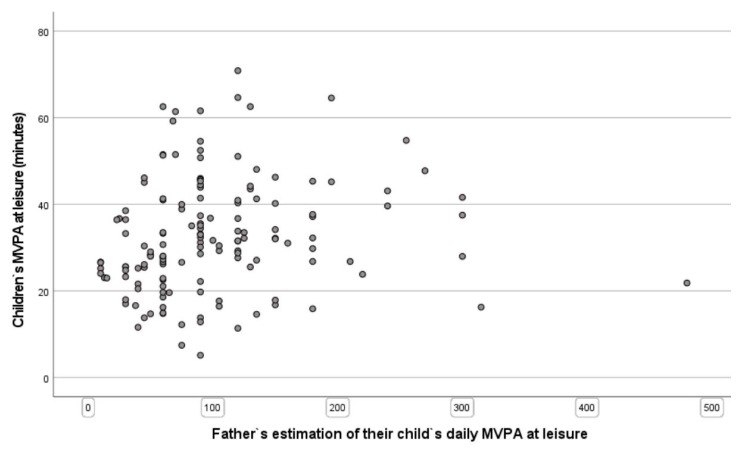
Scatter plot of preschool children’s MVPA at leisure time and fathers’ estimation of their children’s PA level.

In addition, both mothers and fathers overestimated their children’s PA levels. Indeed, according to the objective accelerometer measurements, both mothers and fathers claimed that their children were approximately three times more physically active than in reality. Specifically, mothers and fathers estimated their children to be in MVPA approximately 100 min per day. The results showed that their children’s MVPA was approximately 32 min per day. The great disparity between fathers’ and mothers’ estimation of their children’s PA level and the objective measurements of their children’s MVPA are shown in Figure 1 and Figure 2, respectively.

Only approximately 5% of the parents correctly estimated their children’s activity level. Furthermore, approximately 5% of the parents overestimated their children’s PA levels by more than ten times the objectively-measured activity.

The analyses also showed no significant association between mothers’ and fathers’ reported participation in their children’s PA and their children’s objectively-measured PA (Table 4).

**Figure 3:**
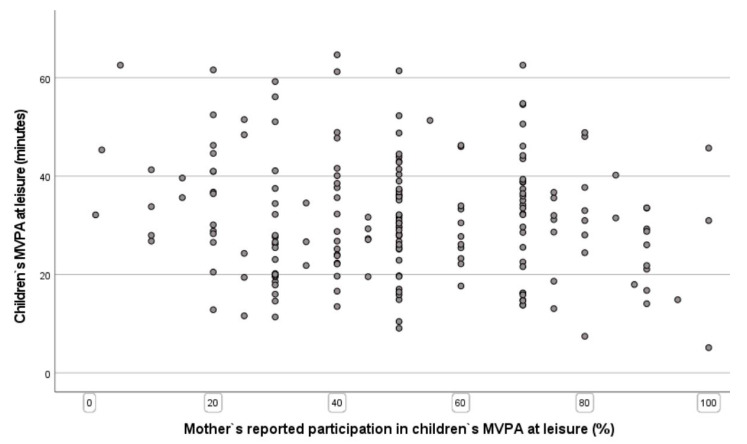
Scatter plot of preschool children’s MVPA at leisure time and mothers’ reported participation in MVPA with their children.

**Figure 4:**
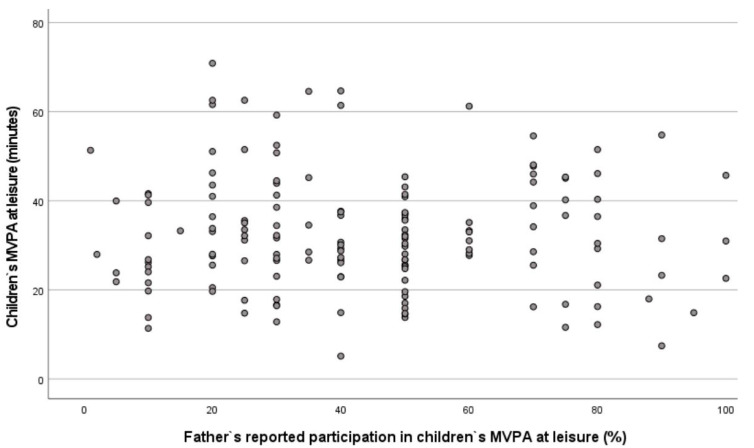
Scatter plot of preschool children’s MVPA at leisure time and fathers’ reported participation in MVPA with their children.

The great disparity between fathers’ and mothers’ parents’ reported participation in MVPA with their children and the objective measurements of their children’s MVPA are shown in Figure 3 and Figure 4, respectively.

On average, the results demonstrate that mothers estimate that they participate in their children’s PA 50.8% of the time, whereas fathers estimate that they participate in their children’s PA 44% of the time.

## 4. Discussion

To the best of the authors’ knowledge, this is the first published study to examine the correlation between parents’ self-reported estimation of their children’s PA and their children’s objectively-measured PA. Furthermore, only a few studies have investigated associations between parents’ participation in their children’s PA and their PA levels. The first main finding was that there was no significant correlation between parents’ estimation of their children’s LTPA and their children’s objectively-measured LTPA. These low associations are visible in the scatter plots; the figures show that parents, in general, overestimate their children’s activity levels and claim that their children are approximately three times more active than they are, according to the objective measurement of the children’s PA. The results of our study are in accordance with The National Institute of Public Health [47], which asserts that self-reporting of PA is significantly overestimated.

Our results show that parents consider their children much more physically active than they are in reality. This phenomenon may lead parents to focus less on their children’s PA than is necessary to influence them to be sufficiently physically active. The consequence may be that children engage in less PA in their leisure time than needed. This phenomenon is unfavorable for children’s total PA per day because research demonstrates a positive relationship between PA in leisure time and preschool [15]. Regarding children’s PA, our study suggests that parents need certain criteria to assist them in accurately estimating their children’s PA level. Naturally, LTPA and PA in preschool will help children achieve a total PA level per day that satisfies the international health recommendations [6]. Zecevic, Tremblay, Lovsin, and Michel [48] find that parents’ assessment of their children’s level of PA may depend on their perception of their children’s level of development (younger children, who typically require more supervision and care, might be perceived as more active), and their perception of their supportive behavior of PA, including their level of PA. This finding is consistent with Corder et al. [26], who highlights that the parental burden of providing support, including transport to PA locations, may lead parents to assume that their child is sufficiently active even if the child does not engage in sufficient PA to meet the PA guidelines. This may constitute a misinterpretation of stress, which leads to an overestimate of the children’s activity because parents’ stress is included in the interpretation of the children’s activity. One possible reason it is so difficult to assess the PA level in one’s own children, according to objectively-measured PA, is that PA intensity is a measurement based on counts converted into the interpretable physiological variable MVPA. Parents may need more specific assessment criteria instead, e.g., movements that make children warm, sweat, and breathe harder. Such movements could include brisk walking, jumping, running, and rough and tumble play, such as wrestling. The use of such appropriate criteria in connection to observation during measurement could be markedly beneficial. Preschools and public health authorities also play a key role in providing information and guidance to parents regarding the major health benefits of being physically active and how to best support their children’s PA. This may include being physically active with their children in activities that, again, make the children warm, sweat, and breathe harder.

The second main finding is that there is no significant correlation between parents’ reported participation in PA and their preschool children’s PA level at leisure time. This result applies to both the mother and the father. This finding is interesting and might imply that children of preschool age are initially physically active, as suggested in another study [49], and that early socialization mechanisms may be more prominent later on. It is also appropriate to point to the limitations of parents’ self-reporting data, with a limited ability to remember the exact amount and intensity of PA and less accurate and more overestimated PA [37,50]. In fact, extant literature reports that adults have a positive impact on preschool children’s PA level [27,51,52], especially those who show enjoyment of PA [34]. This may indicate that parents’ behavior and actions are important factors associated with young children’s activity level. Dowda et al. [29] showed that parental role modelling of PA was not directly related to children’s MVPA. However, even if parental modelling may not affect child PA directly, one can assume that physically active parents are likely to participate in PA to a greater extent with their children and to support their children’s PA. Pfeiffer et al. [53] included the dependent variable of parents’ participation with their children in PA in the variable of family support. Their study found that family support for PA was correlated with non-sedentary activity. According to Corder et al. [26], parents’ participation can reduce the overestimation of the children’s PA level. This is because the perception of stress, in connection with the transport to and from activities, makes the parents think that their child is more physically active than they are in actuality.

According to research, parents must be encouraged to be physically active with their children to improve their children’s overall health. Indeed, previous literature demonstrates that mothers are important role models for PA and that fathers’ reward (praise for participation in PA) and direct support (bringing to and from activities, payment for participation and equipment) are the factors that affect PA most positively [30]. Parents must also be provided with effective guidance on how to facilitate their children’s PA. According to Corder et al. [26], a potential strategy might be to encourage parents to consider whether their children’s activities are sufficiently ‘active’ to meet health guidelines. This finding also supports the promotion of active travel, where children, e.g., walk or ride a bike to school or activities outside school, perhaps as an alternative to parents providing motorized transport in order for children to engage in PA.

It is also essential that parents receive information about the major health benefits of a physically active lifestyle. Preschools and public health authorities serve a key function in providing such information and guidance to parents. This information and support can be provided, for example, through conversations with the parents in preschool or health examination settings. Other complementary intervention strategies include goal setting and personalized feedback to parents about their children, improving parental awareness, and increasing their children’s PA. It is also crucial that preschool staff inform parents about their children’s PA level because studies find that children’s PA follows the same pattern in preschool as in leisure time [54,55]. Author [15] demonstrate that a positive association exists between PA in MVPA at leisure and PA in MVPA at preschool, in which PA in MVPA at preschool increases when PA in MVPA at leisure increases. This will promote awareness of the importance of PA for obtaining major health benefits. In collaboration, preschool, health authorities, and parents can also prevent health inequalities through information about the health benefits of being physically active and facilitating PA in the children’s neighborhoods at leisure. Settings for such facilitation could be safe areas without busy roads and green areas where families can go for walks and recreational outings. Areas for PA in local environments lower the threshold for PA and can contribute to more people becoming physically active. Indeed, low-threshold activities in neighborhoods without requirements for equipment and financial expense should also constitute a priority for health authorities. In addition, initial and continuous professional development opportunities for preschool teachers should also be provided on a regular basis, to increase their role in promoting active children during and out-of-school time.

### Strengths and Limitations of the Study

This study possesses several strengths and weaknesses worth mentioning. In terms of strengths, the study has many participants, reflecting the distribution of boys and girls in Norwegian preschools. Different types and sizes of preschools were also included in the study due to being randomly selected, which yields a representative sample. Moreover, the accelerometer is an objective measurement, decreases subjectivity, and eliminates bias, such as social desirability and recall problems [56]. Furthermore, several researchers identified accelerometers as the optimal method to capture PA in free-living situations [46,57]. Regarding the study’s limitations, the questionnaire may not have sufficiently precise questions to assess children’s PA. Observations of concrete movements could provide a better basis for estimating children’s PA than parents’ self-reports. In addition, although accelerometry is considered an optimal measurement when assessing PA in free-living situations, it underestimates activities related to cycling or types of riding vehicles [50], which is relevant because types of riding vehicles are deemed to be an important factor of PA for preschool children [49].

Furthermore, one study from Norway shows that children have different levels of PA in different preschools [15]. This may also affect the children’s PA at leisure. Finally, neither swimming nor other water activities (due to the instruction of no water-contact of the accelerometers) were included in the data analysis, which might lead to an error in the estimation of children’s PA level [15].

## 5. Conclusions

The results of this study demonstrate that no significant correlation exists between parents’ estimation of their children’s activity level at leisure time and the children’s objectively-measured activity level at leisure time. In general, parents overestimate their children’s activity levels by three times compared with the objective measurements of PA. We argue that our findings indicate that parents’ over-estimation of their children’s PA is problematic. Considering that the PA levels of many children are too low to fulfill internationally established health recommendations, parents’ ‘wrong’ perception about their children urgently needs to be addressed and corrected. We suggest teachers and health professionals encourage parents to be physically active with their children by sharing information about the current and potential future health benefits of a physically active lifestyle. Early childhood teachers and public health authorities have a key role in health promotion and guidance for parents, for example, sharing information and guidance during conversations with parents in preschool and health examinations. Such strategies will promote awareness of the health benefits of PA, regardless of socioeconomic status, and address health inequalities. Further research should focus on which factors explain parents’ overestimation of their children’s PA levels.

## Figures and Tables

**Table 1:** Characteristics of parents’ estimation of their children’s MVPA response rate.

Parents’ Response Rate According to MVPA	Boys	Girls	Total
No parents estimated (%)	12.1	11.7	11.9
Only mothers estimated (%)	25.8	30	27.9
Only fathers estimated (%)	12.9	7.5	10.2
Both parents estimated (%)	49.2	50.8	50

**Table 2:** Descriptive characteristics of the participants.

	Mean (SD) 122 Participants without All Data	Mean (SD) 122 with All Data
Mothers’ participation in children’s PA at leisure (% of children’s MVPA)	50.8 (22)	50.2 (22.1)
Fathers’ participation in children’s PA at leisure (% of children’s MVPA)	44 (23.8)	43 (24)
Mothers’ estimation of children’s minutes of MVPA each weekday at leisure	99.7 (63.7)	102.7 (71.1)
Fathers’ estimation of children’s minutes of MVPA each weekday at leisure	98.3 (68.1)	104.3 (72.6)
Children’s objectively-measured MVPA weekdays at leisure (min)	32.3 (12.8)	31.5 (12.8)
Children who met the health recommendations per day (%)	84	82

**Table 3:** Pearson correlations between parents’ estimation of their children’s PA level at leisure time and the children’s objectively-measured PA level at leisure time.

	Children’s MVPA at Leisure Time (r)
Mothers’ estimation of children’s PA level	0.092 (*p* > 0.05)
Fathers’ estimation of children’s PA level	0.158 (*p* > 0.05)

**Table 4:** Pearson correlations between preschool children’s MVPA and parents’ reported participation in MVPA with their children.

	Children’s MVPA at Leisure Time (r)
Mothers’ participation in PA at leisure time with their children	−0.134 (*p* > 0.05)
Fathers’ participation in PA at leisure time with their children	−0.053 (*p* > 0.05)

## Data Availability

The data presented in this study are available on request from the corresponding author. The data are not publicly available due to privacy.
